# ASO Author Reflections: Practice Patterns and Outcomes Among Surgical Oncology Fellowship Graduates Performing Complex Cancer Surgery in the United States Across Different Career Stages

**DOI:** 10.1245/s10434-024-15519-y

**Published:** 2024-05-28

**Authors:** Diamantis I. Tsilimigras, Timothy M. Pawlik

**Affiliations:** Department of Surgery, Division of Surgical Oncology, The Ohio State University Wexner Medical Center and James Comprehensive Cancer Center, Columbus, OH USA

## Past

Surgical oncology fellowships were developed to help equip surgeons with the necessary skill set for independent practice in a field that involves technically challenging cancer operations.^1,2^ To date, none of the available administrative datasets includes information on fellowship training, year of graduation, and years of independent practice for surgical oncologists in practice. In turn, there is a lack of information regarding practice patterns and outcomes of newly graduated surgical oncologists compared with more experienced surgeons. As such, we sought to examine perioperative mortality and serious complications among Medicare beneficiaries undergoing complex cancer surgery by surgical oncology fellowship graduates across different career stages in the United States.^3^

## Present

Between 2016 and 2021, a total of 11,746 Medicare beneficiaries underwent complex cancer surgery (pancreatectomy: 61.2%; hepatectomy: 19.5%; rectal resection: 13.7%; esophagectomy: 5.6%) by 676 fellowship-trained surgical oncologists in the United States. Through manual screening of each individual surgeon record, we identified surgeons who had formally completed a surgical oncology fellowship in the United States and categorized them into three groups according to years of individual practice; early career (1–7 years, *n* = 375, 55.%); mid-career (8–14 years, *n* = 155, 22.9%), and late career (≥15 years, *n* = 146, 21.6%). Patients who were operated on by early career surgeons less frequently had surgery in a metropolitan area (79.8% vs. 82.7%) while more frequently were treated at a Midwestern (24.9% vs. 14.2%) rather than Northeastern institution (20.6% vs. 26.9%) compared with individuals treated by late career surgeons (all *p* < 0.05). After adjusting for patient, procedural, hospital, and surgeon-level factors, surgical oncologists had comparable risk-adjusted serious complications and 90-day mortality rates irrespective of career stage (early career: 13.0% and 7.2%; mid-career: 12.6% and 6.3%; late career: 12.8% and 6.5%, respectively, all *p* > 0.05). Surgical oncologists who completed a fellowship at a currently ACGME-accredited program had comparable outcomes with surgeons who graduated from nonaccredited programs (serious complications: odds ratio [OR] 0.94, 95% confidence interval [CI] 0.74–1.19; 90-day mortality: OR 1.39, 95% CI 0.97–1.98). Female surgical oncologists represented only 17.7% of all surgeons; yet, the odds of serious complications and 90-day mortality were comparable among male and female surgical oncologists across all career stages. Surgeon case-specific volume independently predicted serious complications not only among early career but also among middle and late career surgical oncologists (high vs. low volume; early career: OR 0.80, 95% CI 0.65–0.98; mid-career: OR 0.81, 95% CI 0.66–0.99; late career: OR 0.78, 95% CI 0.62–0.97).

## Future

The incidence of serious complications and 90-day mortality was comparable among early versus middle/late career surgical oncologists performing complex cancer surgery in the United States. Although male and female surgical oncologists had similar outcomes, female surgeons performing complex cancer surgery were largely underrepresented, underscoring the importance of ongoing diversification in the field of surgical oncology. Individual surgeon volume rather than years of independent practice largely determined perioperative outcomes, highlighting the need to maintain surgical volume even among senior surgical oncologists to ensure delivery of high-quality care to cancer patients.
